# Space, Context, Human Capital: A Macro-Micro Perspective on Social Environment and Financial Literacy in Later Life

**DOI:** 10.1093/geroni/igab046.1320

**Published:** 2021-12-17

**Authors:** Yang Li, Jan Mutchler, Edward Miller, Jing Jian Xiao, Reginald Tucker-Seeley

**Affiliations:** 1 Institute of Social Sciences; 2 University of Massachusetts Boston, Boston, Massachusetts, United States; 3 University of Rhode Island, Kingston, Rhode Island, United States; 4 University of Southern California, Los Angeles, California, United States

## Abstract

Despite a large body of literature documenting the association between individual characteristics and financial literacy, our understanding of the impact of macro-environmental conditions on individual financial literacy remains limited, particularly in later life. Drawing from a micro-macro perspective on the social environment and individual processes, we examined the extent to which three state-level contextual characteristics were associated with individual later-life financial literacy in the United States: tertiary educational attainment, poverty prevalence, and Internet penetration. We utilized data from the 2019 Understanding America Study (UAS) for adults aged 50 years or older to assess financial literacy (n=2,930), and data from the American Community Survey to evaluate contextual conditions. The UAS is a nationally representative survey panel supported by the Social Security Administration and the National Institute on Aging. Cross-sectional multilevel regression models were used to examine the hypothesized effects. We found that state-level poverty prevalence was negatively associated with individual financial literacy while state-level Internet penetration was positively associated with individual financial literacy, over and above individual characteristics known to impact financial literacy. No association was found between state-level educational attainment and individual financial literacy after controlling for respondents’ own education. Findings suggest that the social environment may condition financial literacy in later life through exposure to opportunities that promote knowledge acquisition. Interventions to enhance later-life financial literacy may benefit from targeted approaches that take into account the environmental characteristics of their locations of residence.

